# Cyanobacteria as a Platform for Biofuel Production

**DOI:** 10.3389/fbioe.2013.00007

**Published:** 2013-09-26

**Authors:** Nicole E. Nozzi, John W. K. Oliver, Shota Atsumi

**Affiliations:** ^1^Department of Chemistry, University of California Davis, Davis, CA, USA

**Keywords:** cyanobacteria, biofuel, synthetic biology, metabolic engineering, photosynthesis

## Abstract

Cyanobacteria have great potential as a platform for biofuel production because of their fast growth, ability to fix carbon dioxide gas, and their genetic tractability. Furthermore they do not require fermentable sugars or arable land for growth and so competition with cropland would be greatly reduced. In this perspective we discuss the challenges and areas for improvement most pertinent for advancing cyanobacterial fuel production, including: improving genetic parts, carbon fixation, metabolic flux, nutrient requirements on a large scale, and photosynthetic efficiency using natural light.

On October 31, 2011 world population officially hit seven billion, only 12 years after the six billion mark in 1999. The age of exploration and discovery of untapped land and untouched resources is over, humanity must now learn to cultivate and renew energy sources in the same way that we do for food. The ability of biology to renew and reproduce holds great promise for sustainable fuel production if it can be effectively harnessed. Specifically, photosynthetic life self-replicates the intricate machinery needed for capture and conversion of light energy and waste carbon dioxide. We are already utilizing photosynthesis through the fermentation of farm crops for bioethanol production. However, though this is a renewable source of fuel, it competes with cropland (Witcover et al., 2013). What is needed is biological fuel production that functions orthogonally to food production. The use of plant-derived lignocellulose derived from agricultural waste as a source of fermentable sugars is an option favored by many current start-up companies. However, this material consists mainly of heavily cross-linked polymers responsible for plant structure and strength, thus the process for breaking down such material to a form that can be readily utilized by bacteria for fermentation is a highly energy intensive process (Sanderson, 2011).

Alternatively, the use of photosynthetic microorganisms as a platform for biological fuel production has gained considerable popularity as an option that could potentially avoid some of the problems aforementioned (Machado and Atsumi, 2012). As photosynthetic microorganisms directly fix carbon dioxide as their primary carbon source, the need for a source of fermentable sugars as a carbon feedstock for biological fuel production could be eliminated. Eukaryotic algae and cyanobacteria have been the primary organisms of interest for this strategy of fuel production. Both can grow much faster than plants and do not need to be grown on arable land (Dismukes et al., 2008). Furthermore, these organisms are grown submerged in water, which allows for the use of CO_2_ at higher concentrations than that of ambient air (Sheehan et al., 1998) and could potentially allow for the use of concentrated CO_2_ emissions from waste industrial sources. Research on eukaryotic algae has primarily centered on their ability to produce large amounts of lipids for the production of biodiesel (Pate et al., 2011). However despite years of research, eukaryotic algae have yet to realize their industrial potential and synthetic biology techniques for eukaryotic systems remain elusive limiting our ability to improve and diversify these strains (Radakovits et al., 2010). Cyanobacteria, prokarytic organisms, combine of the advantages of both eukaryotic algae, as a photosynthetic microorganism, and *E. coli*, as a tractable and naturally transformable host.

Cyanobacteria have already been engineered to produce a number of different biofuel related compounds (Machado and Atsumi, 2012). In one of the first examples of biofuel production in cyanobacteria, *Synechococcus elongatus* sp. strain PCC 7942 (*S. elongatus*) was successfully engineered to produce ethanol through the addition of a pyruvate decarboxylase and an alcohol dehydrogenase, redirecting carbon from pyruvate (Deng and Coleman, 1999). Cyanobacterial production of ethanol has since been significantly improved (Dexter and Fu, 2009; Gao et al., 2012). Though compatible with current fuel infrastructure as a supplement to gasoline, ethanol serves as a rather poor gasoline substitute due to its hygroscopicity and low energy density. For these reasons efforts have shifted toward longer carbon chain fuels (Table 1). Isobutyraldehyde, an important chemical feedstock for hydrocarbons normally derived from petroleum, was successfully produced in *S. elongatus* by diverting carbon flux from the valine biosynthesis pathway through the addition of a ketoacid decarboxylase, reaching 1.1 g/L in 8 days (Atsumi et al., 2009). Isobutanol, a promising candidate for a gasoline substitute, can easily be obtained from isobutyraldehyde via chemical conversion. Direct biological production of isobutanol from *S. elongatus* was achieved with the addition of an alcohol dehydrogenase reaching 450 mg/L in 6 days (Atsumi et al., 2009). Three heterologous enzymes, acetolactate synthase, 2-acetolactate decarboxylase, and a secondary alcohol dehydrogenase, introduced into *S. elongatus* allowed for the diversion of carbon flux from pyruvate to the production of the chemical feedstock 2,3-butanediol reaching 2.4 g/L in 21 days (Oliver et al., 2013). Other chemicals produced with heterologous biosynthetic pathways from cyanobacteria include 1-butanol (29.9 mg/L) (Lan and Liao, 2012), 2-methyl-1-butanol (200 mg/L) (Shen and Liao, 2012), acetone (36 mg/L) (Zhou et al., 2012), ethylene (∼171 mg/L⋅day) (Takahama et al., 2003; Ungerer et al., 2012), isoprene (0.05 mg/g dry cell⋅day) (Lindberg et al., 2010), and fatty acids (197 mg/L) (Liu et al., 2011).

**Table 1:** **Titers for various biochemicals**.

Compound	Organism	Titer	Reference
Acetone	*Synechocystis* sp. PCC6803	36 mg/L	Zhou et al. (2012)
2,3-Butanediol	*S. elongatus* sp. PCC7942	2.4 g/L	Oliver et al. (2013)
1-Butanol	*S. elongatus* sp. PCC7942	30 mg/L	Lan and Liao (2012)
Ethanol	*Synechocystis* sp. PCC6803	5.5 g/L	Gao et al. (2012)
Ethylene	*Synechocystis* sp. PCC6803	171 mg/L⋅day	Ungerer et al. (2012)
Fatty acids	*Synechocystis* sp. PCC6803	197 mg/L	Liu et al. (2011)
Isobutanol	*S. elongatus* sp. PCC7942	450 mg/L	Atsumi et al. (2009)
Isobutyraldehyde	*S. elongatus* sp. PCC7942	1.1 g/L	Atsumi et al. (2009)
Isoprene	*Synechocystis* sp. PCC6803	50 μg/g dry cell⋅day	Lindberg et al. (2010)
2-Methyl-1-butanol	*S. elongatus* sp. PCC7942	200 mg/L	Shen and Liao (2012)

These successes clearly demonstrate the malleability of cyanobacteria as a chemical production platform. Over the past 15 years we have moved from detection of the first industrial chemicals produced from exogenous genes in cyanobacteria, through a burst of discovery and experimentation with pathways and design in photosynthetic prokaryotes, to our current status of balancing and matching of production to the metabolism of the host (Oliver et al., 2013). In this perspective we seek to highlight the greatest challenges that must be overcome before sustainable biofuel production in cyanobacteria can be fully realized. We will focus mainly on pathway engineering and strain development (Figure 1). Though we will touch on it, the challenges concerning large scale commercialization are largely beyond the scope of this work.

**Figure 1:**
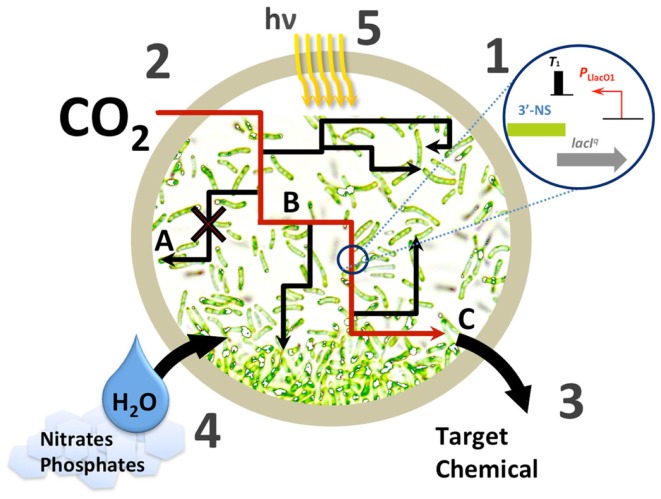
**Challenges in cyanobacterial chemical production**. (1) Improving available biological parts at each level of the central dogma for engineering artificial pathways in cyanobacteria; (2) improving carbon fixation; (3), improving metabolic yield with various strategies, A – eliminating competing pathways, B – improving pathway flux, for example via irreversible steps, C – improving tolerance to or continuous removal of the target chemical; (4), management of limited resources that may be stressed upon scale-up; (5), photosynthetic efficiency and bioreactor design.

## Improving Genetic Parts for Cyanobacteria

Most engineered pathways in cyanobacteria utilize gene expression systems derived from *E. coli* (Atsumi et al., 2009; Shen and Liao, 2012), however it is known that gene expression in *E. coli* cannot accurately predict gene expression in a photosynthetic host (Huang et al., 2010; Oliver et al., 2013). Many engineered systems have employed endogenous cyanobacterial promoters for constitutive expression (Deng and Coleman, 1999), but few such genetic parts have been specifically characterized for utility in metabolic engineering (Heidorn et al., 2011).

The cyanobacterial RNA polymerase (RNAP) holoenzyme contains different subunits than those found in the RNAPs of most bacteria. Furthermore, though cyanobacteria contain σ factors belonging to the σ^70^ family which are also found in *E. coli*, they do not possess any σ^54^ family σ factors which are found in most bacteria (Imamura and Asayama, 2009). With these differences in mind, the discovery that many promoter sequences commonly employed in *E. coli* engineering, such as the lac inducible expression system, do not function well or at all in cyanobacterial systems may not come as a great surprise (Huang et al., 2010; Oliver et al., 2013). Characterization of endogenous cyanobacterial promoters constitutes a short list including light-inducible and metal-inducible promoters (Heidorn et al., 2011). At the translational level, an analysis of ribosomal binding site (RBS) sequences of all genes in *Synechocystis* 6803 revealed that only 26% contained the RBS core sequence compared to 57% in *E. coli* (Ma et al., 2002). Indeed, a comparison of the strength of an RBS sequence modeled after those identified in *Synechocystis* 6803 and three BioBrick sequences further revealed the importance of choosing biological parts adapted specifically for the organism of interest, cyanobacteria in this case (Heidorn et al., 2011). The differences in both transcriptional and translational regulation systems in cyanobacteria must be accounted for if biological parts from *E. coli* are utilized in engineering designs.

Experiments have indicated the operation of anti-sense RNA in regulating gene expression in cyanobacterial systems (Georg and Hess, 2011). Indeed anti-sense suppression has been successfully used in *S. elongatus* to regulate gene expression (Holtman et al., 2005; Cai et al., 2013). Evidence of the CRISPR-Cas system, which has recently become a topic of intense interest, has been identified in most cyanobacterial strains (Cai et al., 2013). The great potential of the CRISPR-Cas system as an engineering tool has already been demonstrated in *E. coli* (Qi et al., 2013).

## Improving CO_2_ Fixation

Despite billions of years of evolution, nature’s ability to effectively sequester CO_2_ would seem on the surface to be strangely inefficient. Ribulose-1,5-bisphosphate carboxylase/oxygenase (RuBisCO), the enzyme responsible for CO_2_ fixation, appears to be lacking in the two qualities enzymes are most known for: high catalytic rate and high specificity (Tcherkez et al., 2006). RuBisCO’s lack of specificity lies in its inherent tendency to bind O_2_ instead of CO_2_, which results in a futile use of energy. Attempts to improve this specificity via site-directed mutagenesis have found that improved specificity always comes at the expense of catalytic rate (Tcherkez et al., 2006; Ninomiya et al., 2008). Consequently, RuBisCO may already be perfectly optimized to the best possible compromise between specificity and catalytic efficiency. Among photosynthetic organisms, cyanobacteria have already evolved very efficient carbon concentrating mechanisms (CCM) which allow RuBisCO to operate near *V*_max_ by lowering the need for high specificity (Savir et al., 2010; Price, 2011).

However, there are large differences between the conditions under which RuBisCO evolved and laboratory or bioreactor conditions which can provide high intensity light and concentrated CO_2_. These artificial conditions may allow for some improvement of carbon fixation. For example, expression of heterologous RuBisCO resulted in a twofold increase in isobutyraldehyde production in *S. elongatus*, indicating a possible increase in carbon fixation (Atsumi et al., 2009). However, the localization of heterologous RuBisCO to the cytosol and not the carboxysome (Iwaki et al., 2006) makes it unclear which limitation is alleviated. Even if we evolve organisms to match saturated CO_2_ conditions, we will still be limited by photosystem II electron turnover after less than a threefold increase in RuBisCO activity (Iwaki et al., 2006).

## Improving Metabolic Yield

Titers for cyanobacterial production of bulk products such as chemical feedstocks, remain significantly lower than fermentative systems (Table 1) (Lan and Liao, 2013). Initial studies reported barely detectable production of chemicals from exogenous pathways (Deng and Coleman, 1999; Lindberg et al., 2010; Lan and Liao, 2011). This has improved rapidly along with host specific design techniques (Atsumi et al., 2009; Ungerer et al., 2012; Oliver et al., 2013). Historically the improvement of exogenous chemical production has followed characterization of the organism. In *E. coli*, arguably the most well characterized model organism available, yields increased steadily along with genetic technology and have started approaching theoretical yields only recently (Inokuma et al., 2010; Shen et al., 2011). *E. coli* still finds little application in industrial settings, due to complications in scale-up (Huffer et al., 2012). Cyanobacteria, while often stated as having a wealth of information available compared to eukaryotic algae, are comparatively uncharacterized for metabolic engineering as compared to fermentative systems (see Improving Genetic Parts for Cyanobacteria). Even on the scale of basic metabolism, a missing gene to complete the TCA cycle in cyanobacteria was only recently characterized (Zhang and Bryant, 2011).

General strategies for metabolic optimization can be grouped into three areas: elimination of competing pathways, maximizing pathway flux, and improving tolerance to or separating out toxic products (with gas stripping for example). Specific to photosynthetic organisms, a fourth strategy can be added to this list: improving carbon uptake, which we discuss in Section “Improving CO_2_ Fixation.” The elimination of competing pathways in cyanobacteria is almost uncharted. Some effort has been put into testing the removal of glycogen pathways (Suzuki et al., 2010), and libraries of knockouts have been constructed (Holtman et al., 2005), however investigations in production optimization similar to those in *E. coli* (Rodriguez and Atsumi, 2012) are hindered by the lack of a strong, standardized chemical production system in cyanobacteria to serve as a benchmark for improvement. In contrast to the strategy of eliminating competing pathways, most work in cyanobacteria has focused on improving pathway flux. For example, production of 2,3-butanediol appears to redirect up to 60% of biomass toward product through the use of irreversible steps, and enzyme screening (Oliver et al., 2013). In the future, computational modeling could be applied to cyanobacteria as it has been to other organisms for the identification of distant pathways that could potentially affect pathway flux, for example in terms of cofactor or ATP availability (Asadollahi et al., 2009; Agren et al., 2013; Misra et al., 2013).

By nature cyanobacteria brings with it an oxygenic atmosphere. This fact automatically limits the list of potential heterologous enzymes available for pathway construction. Many enzymes have displayed lowered activity when transferred into cyanobacteria and have clearly limited production (Lan and Liao, 2012; Ungerer et al., 2012). Changing enzymes in these cases greatly improved yield. Cofactor matching, choosing enzymes that can utilize NADPH versus NADH, is also important when working in a photosynthetic context (Lan and Liao, 2012). Currently pathways exist that appear not to be limited by pathway flux (Oliver et al., 2013), allowing for greater characterization of other limitations in metabolism. Improving tolerance to products should in theory allow for higher titers in culture media (Atsumi et al., 2010), however no investigations into improving cyanobacterial resistance to toxicity have been conducted. Alternatively constant removal of toxic products can increase production (Atsumi et al., 2009; Inokuma et al., 2010; Ungerer et al., 2012), although the feasibility of such processes on an industrial scale remains to be proven.

For continuous production from cyanobacterial strains, culture stability remains a challenge with peak titers occurring after a week in many cases. Loss of production may be due to genetic instability, as carbon diversion creates a selective pressure for spontaneous mutants with an inactive pathway. Production durations have been increased in studies that included codon optimization of key genes to eliminate mutation hotspots, or lowering of end-product toxicity to increase viability of production strains, however more data is needed to clearly define the factors impacting duration (Ungerer et al., 2012; Oliver et al., 2013).

## Managing Inputs: Nutrient and Water Requirements

Though cyanobacteria do not compete with food crops in terms of land, there is some concern that competition for nutrients may become an issue. Cyanobacteria require a source of nitrogen and phosphorous just as plants do, and fertilizer production for plant growth is already a huge industry. Large scale culturing of cyanobacteria with similar nutrient requirements could put significant strain on these resources. A study of potential resource impacts for algae scale-up noted that target production greater than 10 billion gallons per year would require nutrient inputs that could double current fertilizer use (Pate et al., 2011). Current production strains could be expected to give similar nutrient requirement projections.

Phosphate for fertilizer use is sourced entirely from the mining of limited resources. While projections conflict on exactly when these mines will be exhausted, the limitation is clear. We must consider nutrient recovery from domestic wastewater and agricultural runoff. Both biological and chemical methods are being considered for phosphate recovery and recycling. Enhanced biological phosphorous removal (EBPR) utilizes polyphosphate accumulating organisms (PAOs), primarily microorganisms belonging to the *Accumulibacter* group (Yuan et al., 2012). Chemical methods include: chemically reactive filters, enhancing natural struvite (magnesium ammonium phosphate) precipitation, nanomaterials, and polymers with high phosphorous affinity (Pratt et al., 2012). While much work is underway concerning the removal of phosphorous from wastewater, the challenge lies in the recovery of the phosphorous in a way that does not carry along heavy metals and pathogens.

Electricity can be used to generate nitrogen in the Haber–Bosch process, however the bioavailable nitrogen generated in this way is not recycled and can end up as nitrous oxide greenhouse gases. To mitigate this problem renewable strategies for algal recycling of nitrogen have been proposed (Huo et al., 2012). Cyanobacteria capable of efficiently fixing nitrogen from air are known, and possibilities for engineering these strains as production systems remain to be explored (Golden et al., 1985; Steinberg and Meeks, 1991). Large scale cyanobacterial production would also require enormous amounts of water. Current metabolic engineering for chemical production has mainly focused on freshwater strains. To avoid competition with drinking water, large scale production must adapt to strains that can grow and produce competitively in wastewater or salt water. Genetic tools for salt-water strains are available (Frigaard et al., 2004), although no specific exogenous chemical production has been investigated. Work is progressing in the application of eukaryotic algae to wastewater treatment (Cabanelas et al., 2013; Hu et al., 2013), similar work should be applied also to engineered cyanobacteria.

## Photosynthetic Efficiency and Bioreactors

The simple nutrient requirements of cyanobacteria (mainly light, water, and CO_2_) make it an ideal candidate for biofuel production, but the requirement of light exposure makes the design of an industrial bioreactor challenging. To achieve maximum photosynthetic efficiency light must be provided to cells in saturating amounts (Iwaki et al., 2006). Self-shading prevents this from being possible if the culture depth is more than a few inches, and success is highly dependent on the rate of cell mixing (Qiang et al., 1998). Because economy of scale implies longer path lengths (such as raceway ponds) and natural irradiance, industrial outlooks have focused on light as the limiting factor in production calculations. It appears that only 10–15% of sunlight will be available to cells for photosynthesis after non-active radiation, and losses from events such as reflection are subtracted (Robertson et al., 2011). Because of this, maximum production rates measured in high CO_2_ and high light conditions, in particular in strains with improved CO_2_ fixation may not be applicable to large scale systems. New bioreactor designs and production strain designs need to find a balance between capturing all of the available sunlight and using the smallest volume possible to reduce the cost of purification.

The large amount of sunlight incident on cells but not available for photosynthesis is capable of creating heat in cultures. Both this and ambient temperatures may impair production in high insolation areas. The engineering of hyperthermophilic bacteria, capable of tolerating up to 70°C, could alleviate this problem (Onai et al., 2004). Another approach is to convert non-photosynthetically active radiation into usable wavelengths (Wondraczek et al., 2013) to prevent heat absorption.

Industrial systems relying on the natural irradiation of sunlight are limited to a diurnal cycle. In 2013 the successful insertion of sugar transporter systems into *S. elongatus* made it possible for this cyanobacterium to grow in the absence of light (McEwen et al., 2013). This development opens the door to the possibility of 24 hour chemical production by mediating the cost that would be required to electrically illuminate large scale cultures. Cyanobacterial strains that are natural photoheterotrophs such as *Synechocystis* sp. PCC 6803 could also be used for this purpose.

## Final Thoughts

Seen as a list together the challenges to be conquered before cyanobacterial biofuel production can be realized on an industrial scale can seem great. However, taking a step back, the benefits such a renewable system could reap far outweigh the investment necessary to achieve its reality. Significant progress has already been made in each challenge area since interest in cyanobacteria was sparked. The greatest potential to be unlocked lies in the characterization of tightly controlled expression systems tailored for cyanobacteria. Until we can engineer cyanobacteria in a predictable manner, all other concerns, such as those related to scale-up, must wait. “Plug-and-play” biological parts must be adapted to the host of interest. Too long have we been trying to run software designed for a different operating system. Once we are speaking the same language, engineering designs for cyanobacterial systems will balloon with possibilities.

## Conflict of Interest Statement

The authors declare that the research was conducted in the absence of any commercial or financial relationships that could be construed as a potential conflict of interest.
